# Feasibility of Energy Medicine in a Community Teaching Hospital: An Exploratory Case Series

**DOI:** 10.1089/acm.2014.0157

**Published:** 2015-06-01

**Authors:** Francois Dufresne, Bonnie Simmons, Panagiotis J. Vlachostergios, Zachary Fleischner, Ramsey Joudeh, Jill Blakeway, Kell Julliard

**Affiliations:** ^1^ NYU Lutheran Medical Center, Brooklyn, NY.; ^2^ St. George's University School of Medicine, Grenada, West Indies.; ^3^ YinOva Center, New York, NY.

## Abstract

***Background:*** Energy medicine (EM) derives from the theory that a subtle biologic energy can be influenced for therapeutic effect. EM practitioners may be trained within a specific tradition or work solo. Few studies have investigated the feasibility of solo-practitioner EM in hospitals.

***Objective:*** This study investigated the feasibility of EM as provided by a solo practitioner in inpatient and emergent settings.

***Design:*** Feasibility study, including a prospective case series.

***Settings:*** Inpatient units and emergency department.

***Outcome measures:*** To investigate the feasibility of EM, acceptability, demand, implementation, and practicality were assessed. Short-term clinical changes were documented by treating physicians.

***Participants:*** Patients, employees, and family members were enrolled in the study only if study physicians expected no or slow improvement in specific symptoms. Those with secondary gains or who could not communicate perception of symptom change were excluded.

***Results:*** EM was found to have acceptability and demand, and implementation was smooth because study procedures dovetailed with conventional clinical practice. Practicality was acceptable within the study but was low upon further application of EM because of cost of program administration. Twenty-four of 32 patients requested relief from pain. Of 50 reports of pain, 5 (10%) showed no improvement; 4 (8%), slight improvement; 3 (6%), moderate improvement; and 38 (76%), marked improvement. Twenty-one patients had issues other than pain. Of 29 non–pain-related problems, 3 (10%) showed no, 2 (7%) showed slight, 1 (4%) showed moderate, and 23 (79%) showed marked improvement. Changes during EM sessions were usually immediate.

***Conclusions:*** This study successfully implemented EM provided by a solo practitioner in inpatient and emergent hospital settings and found that acceptability and demand justified its presence. Most patients experienced marked, immediate improvement of symptoms associated with their chief complaint. Substantial practicality issues must be addressed to implement EM clinically in a hospital, however.

## Introduction

Energy healing and energy medicine (EM) are terms derived from the theory that a subtle biologic or spiritual energy surrounds and permeates the body and can be influenced for therapeutic effect.^1,2^ Known by various names in 97 different cultures,^3^ the concept of energy healing has been recorded throughout history. The National Institutes of Health includes energy healing therapy in its list of popular complementary and alternative medicine (CAM) methods.^4^

Practitioners of EM treat the patient in close proximity (often with minimal or no physical contact) as well as at a distance (from a different room or even a different time zone). Studies have shown EM to improve pain, anxiety, wound healing, functional status, blood pressure, immune function, relaxation, well-being,^5^ cancer outcomes,^6,7^ fatigue, mood,^2^ fibromyalgia, phantom limb pain, and carpal tunnel syndrome.^8^ No report was found in the published literature of increased mortality, morbidity,^8^ or serious adverse effects,^1^ although some caution in patient selection is advisable.^9^

Even though mechanisms of EM have not yet been established in terms of biomedical science, theories have been advanced,^10^ and EM is increasingly being offered to both inpatients and outpatients by major hospitals. The growth and acceptance of EM have resulted largely from patient satisfaction, with some surveys reporting the percentage of “satisfied users” as high as 98%.^11^

Many forms of energy medicine are practiced by trained practitioners within specific traditions, such as Reiki, Healing Touch, and Therapeutic Touch. In addition, however, some solo practitioners discover their ability to effect positive health status changes and practice EM without or in addition to formal training. Many studies have investigated trained practitioners from various schools,^12,13^ but few have explored how solo practitioners (those unaffiliated with a particular system of EM) can feasibly be integrated into clinical care.

Bowen et al.^14^ suggest that feasibility studies are valuable when few published studies or data exist for a particular intervention and the sociocultural context of an intervention is unclear. Both of these considerations apply to patients and providers at community hospitals with respect to interventions involving solo EM practitioners. Bowen et al. believe that feasibility studies can lay the foundation for more rigorous research of therapeutic interventions by exploring their acceptability, demand, implementation, and integration, among other factors. Investigations for these dimensions of EM are needed to make future research in community hospital settings possible.

The present study investigated the feasibility of implementing EM with a single solo practitioner in the conventional inpatient, outpatient, and emergent settings of a community teaching hospital. Aspects of feasibility examined were acceptability, demand, implementation, and practicality, assessed in part through clinicians' qualitative responses.^14^ The study also documented conventionally recorded clinical changes immediately following EM.

## Methods

This feasibility study and prospective exploratory case series were conducted at Lutheran Medical Center, a full-service community teaching hospital located in Brooklyn, New York. The hospital's institutional review board (IRB) approved the study by expedited review in 4 days. The last author (K.J.) was the administrator of the IRB but was not a voting member or reviewer of the IRB. He was asked to meet with the medical director, the chair of the IRB, and the vice president for professional affairs to answer questions about EM, the practitioner, and the study before approval.

A solo EM practitioner with 14 years of experience who had recently seen patients at the YinOva Center, a holistic wellness center in Manhattan, provided the EM sessions. The director of the YinOva Center (J.B.) founded the inpatient acupuncture program at Lutheran Medical Center in 2003 and was a trusted colleague. The decision to work with the EM practitioner and conduct the study was based on the director's experience with and knowledge of his work. While Lutheran Medical Center had osteopathic residency programs and an osteopathic medical school onsite, was oriented toward whole-person care (body, mind, spirit, and community), and was open to CAM,^15^ no CAM or EM programs other than osteopathy and acupuncture existed at the time of the present study.

The EM practitioner was oriented to the medical center by the Volunteer Department, through which he was processed. The last author (K.J.) approached the physician unit leader and head nurse manager of three patient centers in the hospital to obtain permission to conduct the study on their units. Permission was granted for all three, after which this author conducted a brief in-service training for the unit's physicians, nurses, and allied health staff. The forms of energy medicine and the process of the study, including inclusion and exclusion criteria, were explained in a session lasting 15 to 30 minutes, depending on questions. The process of approaching the units and obtaining approval took about 2 weeks.

After the training, any health professional on the unit was eligible to identify a patient for potential inclusion, a process that took an additional week and required personal encouragement by the last author to initiate. The EM practitioner was supervised by attending physicians, residents, and nurses in study locations. Study physicians and the patient's attending physician approved each patient's participation and verified inclusion and exclusion criteria. Approved patients were approached by a member of the study team to obtain informed consent. Outpatients and nonpatients were evaluated and enrolled in a similar way.^9^

Using their clinical judgment, physicians identified as potential participants adult patients, employees, or friends or family of employees who had signs and symptoms that were not responding to traditional medical therapy or were only slowly responding. Patients deemed to have secondary gains for their medical condition or who were unable or unwilling to communicate with the research team regarding the effects of the energy medicine session were excluded from the study. Assessments by which improvement was typically gauged in this clinical setting were specified for each individual patient on the basis of the judgment of his or her treating physician.

During each session, the solo EM practitioner was accompanied by a research team member and usually by other hospital staff already working with the patient, such as a nurse. Pretreatment assessments were made and recorded by a research team member with respect to the patient's expressed chief complaint. The EM practitioner was introduced to the patient and inquired about symptoms and goals for treatment, sitting at the bedside or in proximity to the patient. He sometimes positioned his hands over the affected area. No physical contact occurred between him and the patient. This generally lasted for a minute or two at a time, allowing the practitioner to receive ongoing feedback from the patient. He repeated the process several times as needed to address different problem areas, adjusting his method on the basis of information supplied by the patient. For some patients, the practitioner “energized” water that the patient then drank. This was done as follows. While with the patient, the practitioner placed his hands in proximity to a cup of water already in the patient's room and mentally directed energy to it without touch, in the same way as with the patient. The patient would then drink the water.

At the session's conclusion, post-treatment assessments were recorded by the research team; these assessments of pain and other clinical indicators were carried out according to the hospital's standard of care. Some patients received additional sessions as reported in the tables, depending on improvement, availability, and the patient's wishes.

Summary descriptive statistics were created for two subgroups of patients: those with complaints of pain and those requesting help with symptoms or signs not related to pain. Improvement of pain was rated as none (no change), slight (pain scale improvement of 2 points or less, or qualitative rating only), moderate (pain scale improvement of 3–5 points), and marked (improvement of 6 points or more). Pain that resolved completely (pain scale score, 0 of 10) was also classified as a marked improvement. Improvement of symptoms and signs other than pain was also rated on a scale of none, slight, moderate, and marked. The system by which standard assessments in our setting were converted into this rating scale was developed by consensus of all authors.

Acceptability of EM was investigated by determining whether physicians would recommend patients for the study and whether patients would accept such treatment. Demand was investigated by recording the complaints for which patients and physicians requested EM sessions. Implementation was investigated by assessing whether the study's in-service and referral system resulted in a manageable number of sessions. Practicality was investigated by assessing the resources, time, staffing, and credentialing needed to carry out the study.

The chi-square or Fisher exact test was used to determine significance of differences between assessments of change by subgroup of demographic and clinical characteristics.

## Results

**FIG. 1.**
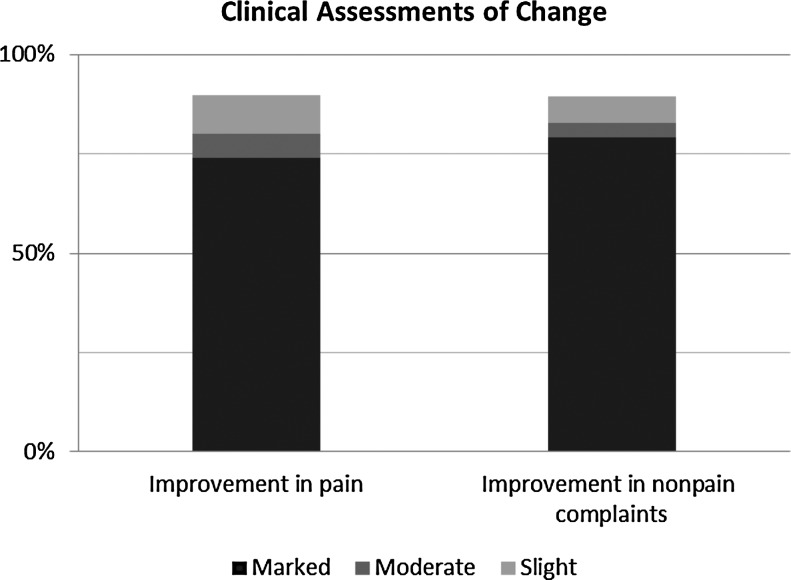
Percentage of assessments of improvement in patients with pain and nonpain complaints.

Thirty-two patients were treated with EM as part of the study. The 24 patients who requested relief from pain had a mean age of 55.5 years (range, 25–87 years). Four (17%) were male and 20 (83%) were female. Eighteen (75%) were inpatients, and 6 (25%) were outpatients or employees (Table 1). Of the 50 individual reports of pain, 5 (10%) showed no improvement; 4 (8%), slight improvement; 3 (6%), moderate improvement; and 38 (76%), marked improvement (Fig. 1).

**Table 1.** Results of Energy Medicine with Pain

*ID*	*Age (yr), sex, inpatient or outpatient status*	*Diagnoses*	*Problems assessed*	*Before EM*	*After EM*	*Sessions/days of follow-up*
1	86, female, inpatient	Urinary tract infection	Suprapubic pain	Pain 10/10	Pain 0/10	1/0
2	57, male, inpatient	Osteoarthritis Prostate cancer	Right hand pain	Right hand pain 9/10	Right hand pain 9/10	3/1
2	57, male, inpatient	Osteoarthritis Prostate cancer	Right knee pain	Right knee pain 9/10	Right knee pain 9/10	3/1
3	33, female, inpatient	Syncope Disc herniation Hypertension	Lower back pain	Lower back pain 8/10	Lower back pain 0/10	2/1
3	33, female, inpatient	Syncope Disc herniation Hypertension	Right lower abdominal pain	Right lower abdominal pain 6/10	Right lower abdominal pain 0/10	2/1
4^a^	58, male, inpatient	Peripheral vascular disease with right above-knee amputation	Right phantom limb pain Left leg pain	Right phantom limb pain 10/10 Left leg pain 10/10	Right phantom limb pain 0/10 Left leg pain 8/10	2/1
5^a^	62, female, inpatient	Cholecystitis	Epigastric pain Left shoulder pain Right abdominal pain Back pain	Epigastric pain 10/10 Left shoulder pain 10/10 Right abdominal pain 10/10 Back pain 10/10	Epigastric pain 0/10 Left shoulder pain 0/10 Right abdominal pain 0/10 Back pain 0/10	2/1
5^a^	62, female, inpatient	Right pleural effusion	Epigastric pain Left shoulder pain Right abdominal pain Back pain	Epigastric pain 10/10 Left shoulder pain 10/10 Right abdominal pain 10/10 Back pain 10/10	Epigastric pain 0/10 Left shoulder pain 0/10 Right abdominal pain 0/10 Back pain 0/10	2/1
6^a^	87, female, inpatient	Coronary artery disease Diverticulosis with bowel resection	Left arm pain	Left arm pain	Left arm pain 0/10	1/0
6^a^	87, female, inpatient	Coronary artery disease Diverticulosis with bowel resection	Neck pain	Neck pain	Neck pain 0/10	1/0
7^a^	34, female, inpatient	Cervical cancer	Lower abdominal pain	Lower abdominal pain 10/10	Lower abdominal pain 0/10	2/1
8	70, female, inpatient	Ischemic colitis Disc herniation	Lower back pain	Lower back pain 8/10,	Lower back pain 0/10	2/1
9	76, female, inpatient	Left leg pain secondary to right leg cellulitis	Left leg pain	Left leg pain 10/10	Left leg pain 3/10	1/0
11^a^	56, female, outpatient (emergency department visit, not admitted)	Migraine associated with unilateral motor symptoms Conversion disorder	Lower back pain	Low back pain 8/10	Low back pain 5/10	1/0
11^a^	56, female, outpatient (emergency department visit, not admitted)	1. Migraine associated with unilateral motor symptoms 2. Conversion disorder	2. Neck pain	Neck pain 5/10	Neck pain “improved”; patient did not specify further	1/0
12	Female, employee	No past medical history	Right hip pain	Right hip pain 4/10	Right hip pain 0/10	1/0
13	45, female, employee	Left frozen shoulder	Pain upon left arm elevation over 90 degrees	Pain upon left arm elevation over 90 degrees 6/10	Pain 0/10	1/0
14^a^	34, female, inpatient	Acute myeloid leukemia	Lower back pain	Lower back pain 10/10	Lower back pain 0/10	3/2
14^a^	34, female, inpatient	Acute myeloid leukemia	Stomach pain	Stomach pain 7/10	Stomach pain 7/10	3/2
14^a^	34, female, inpatient	Acute myeloid leukemia	Bilateral knee pain	Bilateral knee pain 7/10	Bilateral knee pain 0/10	3/2
15	25 female, inpatient	Peptic ulcer disease Gastroparesis Diabetes mellitus	Back pain	Back pain 10/10	Patient states that she is “still in a lot of pain”; did not specify further	1/0
15	25, female, inpatient	Peptic ulcer disease Gastroparesis Diabetes mellitus	Stomach pain	Stomach pain 10/10	Patient states that she is “still in a lot of pain”; did not specify further	1/0
17	61, female, inpatient	Coronary artery disease Congestive heart failure	Right-sided neck pain	Right-sided neck pain; patient could not turn her head toward right side	Complete relief in her neck pain and ability to turn the head to the right without effort	2/1
17	61, female, inpatient	Coronary artery disease Congestive heart failure	Chest pain	Chest pain on deep inspiration	No chest pain with inspiration	2/1
19	56, female, inpatient	Chronic back pain Depression	Pain in right hand	Pain right hand 8/10	Pain right hand 7/10	3/1
19	56, female, inpatient	Chronic back pain Depression	Pain in fingers of right hand	Pain right hand with shaking right hand	Pain with shaking “1 degree down”; patient did not elaborate further	3/1
19	56, female, inpatient	Chronic back pain Depression	Pain in left hand	Pain in left hand 10/10	Pain in left hand 7/10	3/1
20	33, male, inpatient	Coronary artery disease	Chest pain	Pain 7/10 Pain 7/10 not changed by active range of motion of left arm Left frontal wall chest pain radiating to left shoulder	Pain 1–2/10 immediately after session, 1 hour later pain 2–3/10, 1 day later pain 7/10 but limited to same small area Pain 1–2/10 on full active range of motion of left arm Area of pain decreased from entire left frontal chest wall to a small linear vertical area of left upper arm, giving a pinch sensation rather than actual pain, 1 day later pain limited to same small area	5/1
21^a^	67, female, inpatient	Acute respiratory failure from pneumonia (patient was intubated) Chronic obstructive pulmonary disease	Abdominal pain	Abdominal pain 8/10	Abdominal pain 1/10	4/1 (via PEG tube while patient was still intubated but awake)
21^a^	67, female, inpatient	Acute respiratory failure from pneumonia (patient was intubated) Chronic obstructive pulmonary disease	Low back pain	Low back pain 8/10	Low back pain 0/10	4/1 (via PEG tube while patient was still intubated but awake)
22	50, male, outpatient	Kidney stone	Renal colic from kidney stone	Renal colic 7/10	Low cramping, “poking” 0.5–1/10 pain for 3 weeks following episode, then 0/10 of pain after that time	2/1
23	63, female, inpatient	Mechanical fall Fracture lumbar spine	Left leg pain	Left leg pain 5/10	Left leg pain 0/10	1/0
23	63, female, inpatient	Mechanical fall Fracture lumbar spine	Lower back pain	Lower back pain 5/10	Lower back pain 2/10	1/0
23	63, female, inpatient	Mechanical fall Fracture lumbar spine	Right leg pain	Right leg pain 5/10	Right leg pain 0/10 (Patient was re-evaluated the next morning and the similar degree of relief was maintained)	1/0
24	72, female, inpatient	Osteoarthritis bilateral knees	Chronic bilateral knee joint pain	Bilateral knee pain 10/10	Pain 0/10 bilateral knees	1/0
29^a^	55, female, employee	Migraine headaches	Migraine headaches	Headache 10/10 Headache 6/7 days per week	Headache 0/10 Headache 1.5/7 days per week (periods of 1.5 week with no headache)	10/35
30	52, female, employee	Chronic lower back pain	Chronic lower back pain	Chronic lower back pain	Complete resolution of chronic lower back pain	1/0
30	52, female, employee	New-onset lower back pain	New-onset lower back pain	New-onset lower back pain	Complete resolution of new onset lower back pain	1/0
31	45, female, inpatient	Breast cancer metastatic to the liver	Right upper abdominal pain	6/10	1. 0/10	1/0 (pain relief sustained 15 minutes after treatment)
31	45, female, inpatient	Breast cancer metastatic to the liver	Lower back pain	7/10	2. 0/10	1/0 (pain relief sustained 15 minutes after treatment)

^a^ Patient drank charged water as part of energy medicine session.

EM, energy medicine; PEG, percutaneous endoscopic gastrostomy.

Twenty-one patients had a wide variety of issues other than pain (Table 2). Their mean age was 59.9 years (range, 22–87 years). Eight (38%) were male, and 13 (62%) were female. Seventeen (81%) were inpatients, and 4 (19%) were outpatients or employees. Of the 29 non–pain-related symptoms and signs, 3 (10%) had no, 2 (7%) had slight, 1 (4%) had moderate, and 23 (79%) had marked improvement (Fig. 1). Assessments of change did not differ by sex, age, location of symptoms, use of charged water, or severity of symptoms in either the pain or the nonpain group (*p*>0.05). The accompanying physicians noticed that when change took place during an EM session, that change was immediate.

**Table 2.** Effect of Energy Medicine on Symptoms Other Than Pain

*ID*	*Age (yr), sex, inpatient or outpatient status*	*Diagnoses*	*Problems assessed*	*Measurements*	*Before EM*	*After EM*	*Sessions/days of follow-up*
1	86, female, inpatient	Asthma exacerbation	SOB	Presence/absence of SOB	Severe SOB	No SOB	1/0
2	57, male, inpatient	Osteoarthritis Prostate cancer	Inability to flex fingers	Passive range of motion	Inability to flex fingers	Improvement in flexion of fingers	3/1
3	33, female, inpatient	Syncope Disc herniation Hypertension	Inability of hip flexion	Passive range of motion	Inability of hip flexion	90 degrees hip flexion	2/1
6^a^	87, female, inpatient	Coronary artery disease Diverticulosis with bowel resection	Dysphagia	Presence/absence of dysphagia	Dysphagia	No dysphagia	1/0
7^a^	34, female, inpatient	Hematuria	Hematuria Burning urination Urgency Dysuria	Presence/absence of symptom	Hematuria Burning urination Urgency Dysuria (pain in urination)	No hematuria No burning urination No urgency No dysuria	2/1
8	70, female, inpatient	Ischemic colitis Disc herniation	Bloody diarrhea	Presence/absence of bloody diarrhea	Bloody diarrhea 6 times/day	No bloody diarrhea	2/1
10	62, male, inpatient	Infected right leg wound after bypass graft surgery	Fever Infection	Presence or absence of fever Resolution time of infection	Fever Infection	No fever Rapid recovery from infection	1/0
11^a^	56, female, outpatient (emergency department visit, not admitted)	Migraine associated with unilateral motor symptoms Conversion disorder	Right hemiparesis	Presence/absence of symptom	Right hemiparesis	Right hemiparesis unchanged	1/0
11^a^	56, female, outpatient (emergency department visit, not admitted)	Migraine associated with unilateral motor symptoms Conversion disorder	Slurred speech	Presence/absence of symptom	Slurred speech	Slurred speech improved	1/0
13	45, female, employee	Left frozen shoulder	Pain upon left arm elevation over 90 degrees	Active range of motion	Active range of motion 90 degrees	Maximum active range of motion of left arm went from 90 to 120 degrees without pain	1/0
16^a^	70, female, inpatient	End-stage metastatic colon cancer	Nausea Vomiting	Presence/absence or symptom	Nausea with consumption of food or drink Vomiting up to 6–8 times daily	Patient was able to drink water without nausea immediately post-session Only 1 episode of vomiting from the time of initial evaluation (approximately 15 hours earlier)	1/0
21^a^	67, female, inpatient	Acute respiratory failure from pneumonia (patient was intubated) Chronic obstructive pulmonary disease	Weaning	Success of weaning trials	Weaning trial (previously unsuccessful 1 day before)	30%–40% improvement of weaning effort on day 1 of session; successful weaning 1 day after EM session	4/1 (via PEG tube while patient was still intubated but awake)
22	50, male, outpatient	Nasal congestion	Nasal congestion	Presence/absence of symptom	Nasal congestion	No congestion immediately after session	2/1
24	72, female, inpatient	Osteoarthritis bilateral knees	Reduced active range of motion	Degree of active range of motion Degree of passive range of motion	No active range of motion bilateral knees, patient unable to walk due to pain 50 degrees left knee passive range of motion; no passive range of motion right knee	Full active range of motion bilateral knees, patient stood up and walked with cane; 90 degrees passive range of motion bilateral knees	1/0
25	66, female, inpatient	Sepsis from superficial necrotizing fasciitis	Severe sepsis from superficial necrotizing fasciitis	Survival (100% preoperative mortality, 60% postoperative mortality) Clinical improvement (patient was intubated but awake)	100% preoperative mortality, 60% postoperative mortality	Patient survived after surgical debridement and antibiotic treatment Patient improved clinically and was sent to short-term rehabilitation facility	1/0
26^a^	60, male, inpatient	Parkinson's disease	Joint stiffness, reduced mobility-walking-getting from chair to bed ability	Presence or improvement/absence of symptom	Joint stiffness, reduced mobility-walking-getting from chair to bed ability	Patient felt “a little bit better” when he stood up and used hands-wrists, was able to take a few steps, 10%–20% improvement	1/0 (remote phone session)
26^a^	60, male, inpatient	Parkinson's disease	Muffled speech, difficulty in word articulation	Presence or improvement/absence of symptom	Muffled speech, difficulty in word articulation	No change in speech	1/0 (remote-phone session)
27^a^	76, female, inpatient	Left leg gangrene	Left leg gangrene	Presence or improvement/absence of condition/sign	Left leg gangrene	No resolution of left leg gangrene	1/0
27^a^	76, female, inpatient	Left leg gangrene	Fever	Presence or improvement/absence of condition/sign	Fever	Absence of Fever	1/0
28	22, female, outpatient	IgE–mediated food allergies (including peaches, melon, strawberries, apples, almonds and soy)	Patient was instructed to eat a small amount of strawberry and was observed for possible allergic reaction	Presence or improvement/absence of allergy symptoms (shortness of breath, skin rash)	History of food allergies; exposure to strawberry (known allergen for patient)	No allergic reaction after exposure to known allergen	3/38
32	53, male outpatient	Herpes simplex of the tongue and lip	New lesion	Painful tingling Visible lesion	10/10 Dark red vesicle	0/10 Vesicles became smaller immediately, complete resolution 48 hr	2/2

^a^ Patient drank charged water as part of EM session.

SOB, shortness of breath.

Regarding acceptability and demand (Tables 1 and 2), physician referrals came largely from a small group of early adopters on each unit and, within 2 weeks of the first in-service, training met the capacity of the single EM practitioner. Most patients were favorable to EM once approached. Those who declined EM did so for various reasons: religious beliefs, pain so intense they did not want any interaction, “not wanting to be bothered,” or a conviction that it would have no benefit (nonbelief). Most patients who found improvement exhibited both relief and surprise, to varying degrees. Some felt disappointment after an unsuccessful attempt, but most patients in whom the intervention was unsuccessful were neutral, perhaps an indication of low pre-intervention expectations.

Implementation of EM was smooth. The study team found no significant change introduced by the EM sessions in their routine medical practice because it dovetailed with conventional goals of care and clinical assessments of progress. A few referring physicians commented that the speed of recovery was enhanced in patients who perceived positive clinical results; most did not inquire about the outcome. Regarding practicality, the study was practical in our setting because it was time limited, relied on assessments physicians typically make, and was staffed on a volunteer basis. The resources determined from carrying out the study that would be needed to implement EM as a formal program in the hospital were not available on the hospital's tight operating budget; thus, this EM program was not practical in our setting outside the study.

### Patient example 1

Patient 31 was a 45-year-old woman with a diagnosis of metastatic breast cancer since 2003. Given the progression of her disease, pain became a major morbidity. On this admission, she presented with severe upper abdominal pain that had worsened in the previous 2 days. She reported that pain intensified with movement or touching of the affected area. During EM, she lay on her bed. In the room were two medical attendings and the EM practitioner. On initial assessment, the patient stated that her abdominal pain was improved to a score of 6 of 10 since admission but that she had significant (7 of 10) mid-lower back pain. The practitioner placed his hands approximately 10 inches above her right upper abdomen for approximately 20 seconds without touching her. Immediately afterward, she rated the abdominal pain to be 0 of 10. He addressed her lower back by placing his hands several inches over her umbilicus, after which she reported a pain score of 0 of 10. The practitioner asked her about the location of the cancer and did further work on the liver area. The patient was reassessed 15 minutes later and reported a sustained relief from pain in both areas.

### Patient example 2

Patient 24 was a 72-year-old woman who presented to the emergency department with bilateral worsening knee pain, inability to walk, and inability to bend her knees. She had been told that she needed bilateral knee replacements, but her cardiac status contraindicated surgery. The patient arrived with her husband, who was sympathetic to her pain and frustrated by her inability to ambulate and the ineffectiveness of her pain medication. Before EM, her pain was 10 of 10 in both knees. She had less than 5 degrees of active range of motion. Passive range of motion was 3.5 degrees in the left knee and 0 degrees in the right knee. After the session, her pain was 0 of 10, and she had full active range of motion in both knees. She stood up and walked with a cane, smiling. Her husband applauded and said that he hadn't seen her do this in many years.

## Discussion

This study found that integrating solo-practitioner energy medicine into inpatient and emergent hospital settings was largely feasible within the parameters of the study itself. In addition, it found significant immediate improvements in most patients after EM sessions with respect to symptoms of their medical conditions and, to a lesser extent, with signs. Before the sessions, physicians identified these signs and symptoms as in their judgment unlikely to change rapidly.

### Comparison of findings

Published reviews of research on biofield healing^16^ and EM^13,17–19^ suggest a measurable benefit of EM with pain. Although the current clinical assessments of improvement are not directly comparable given the differences in study population and the EM modality used (solo practitioner), a clinically significant reduction of pain occurred in most of our patients. With respect to musculoskeletal and arthritic pain and limitation of movement, several studies^20–23^ suggest that function and range of motion can be improved along with pain. The current results concur with theirs.

Studies of the effectiveness of EM or biofield therapies on cancer-related symptoms (pain, fatigue, anxiety, and depression) reported a trend toward improvement.^2^ However, the evidence for the effectiveness of biofield therapies in reducing fatigue and enhancing quality of life in these patients is still inconclusive.^24^ The current study included only five patients with cancer but suggests that EM can be of benefit in immediate relief of symptoms.

### Considerations regarding feasibility

The present study provides information concerning how EM may feasibly be integrated into a hospital setting. Reiki programs in hospitals have often been staffed by volunteers and sometimes by professionals.^13,21,25^ For example, volunteer Reiki practitioners have been involved in the management of anxiety, pain, and global wellness in patients with cancer.^26^ Sessions were felt helpful in improving well-being, relaxation, pain relief, sleep quality, and reducing anxiety of patients attending an outpatient oncology and infusion services unit. This suggests that solo-practitioner EM in hospitals could benefit patients' physical and emotional needs.

Because the EM practitioner in our study was flexible regarding timing and location of sessions, it was logistically easy to provide sessions that did not interfere with traditional treatment. Further, the patients' treating physicians noted no evidence of adverse effects associated with EM at the time of final assessment after EM. The ethical principles of patient autonomy and non-maleficence, along with the lack of adverse effects and ease of integration of EM, encourage further use of this modality in the hospital setting.^13^ Hospital staff had a wide range of opinions related to EM, but as evidenced by the volume of referrals to the EM practitioner, staff members who might not have otherwise recommended EM to patients did so. Given the popularity of CAM with patients, physicians might be persuaded to allow inpatient EM as being preferable to unknown CAM practices done outside of their supervision.

Issues related to clinical implementation encountered in designing and carrying out this study were as follows: (1) credentialing and status of the EM practitioner; (2) administrative approval, quality monitoring, and consent processes; (3) physician, nurse, and patient acceptance; and (4) payment. The following section briefly discusses each of these issues.

First, because no form of EM with the exception of acupuncture is currently licensed, hospital credentialing may not be possible for other EM practitioners, even when associated with a specific school. Hospitals or clinics often offer Reiki with volunteers who have undergone the clinical institution's specific orientation, who are supervised by nurses and have the approval of physicians whose patients will receive Reiki. Since Therapeutic Touch and Healing Touch as forms of EM are generally considered to be within nurses' scope of practice, this is considered appropriate, and thus nurses may be able to supervise solo practitioners as well. As an official Lutheran Medical Center volunteer, the solo EM practitioner in the current study was covered by the hospital's general liability insurance; he made no physical contact with patients and so credentialing was not an issue. An extra layer of administrative approval would be needed if EM were provided outside of an IRB-approved research study, however.

Second, even a volunteer EM program is not without cost, given the logistics of planning the program; enrolling, training, and supervising practitioners; and managing patient referrals. Thus, obtaining administrative approval is a critical and often thorny step for solo EM practitioners, more so than for those trained by specific schools. The EM program and its individual practitioners need to be monitored for quality, in both their adherence to program guidelines and clinical benefit, harm, and patient satisfaction (the tables in our study suggest ways of assessing benefit). Part of designing an EM program includes how consent will be handled. Each health system will need to decide whether a specific consent is obtained from the patient or whether the institution's overall consent is considered sufficient. State regulations play a role in this as well.

Third, physician, nurse, and patient acceptance of EM sessions are equally important. Without physician and nurse acceptance, patients are unlikely to know about the service. In addition, patients' level of pain, religious beliefs, and worldview may block EM as an option they will consider. Approaching patients with sensitivity to these issues is important. Hospital chaplains may be able to assist with this process.

Finally, a skilled EM practitioner may not be willing to offer services on a volunteer basis, although that was the case in the present study. Payment options will vary widely depending on the institution. A nurse could feasibly supervise an EM solo practitioner who receives compensation.

### Limitations and strengths

The limitations of this study mostly follow from the fact that EM research is complex and developing, with answers to key questions of mechanism, measurement, efficacy, and effectiveness still evolving. This limited the dimensions of feasibility that this study could investigate. Regarding the assessments of clinical benefit, the limitations are similar to those of any case series reporting on an innovative technique of treatment: unclear generalizability to a larger population with the same medical issues and difficulty in establishing reliable estimates of benefit. Furthermore, because the mechanism of EM is poorly understood, it is difficult to compare individual practitioners as to whether they will achieve similar results. Even with these limitations, the improvement patients experienced was often striking, providing justification for developing a clinical protocol whereby some solo EM practitioners could be integrated into clinical care after a period of assessment. It also suggests possibilities for how to document clinical assessments related to treatments given by solo EM practitioners and highlights the value of case series in exploring new techniques.^27^

Previous studies on Reiki and other forms of energy healing sometimes involved physical contact between the healer and patient. With the possible exception of a handshake, no physical contact between the EM practitioner and the patient occurred in this study. A 2013 study on therapeutic massage featuring touch and no-touch controls found a reduced placebo effect with the no-touch control, suggesting that the lack of contact in the present study strengthens the results and diminishes the placebo effect.^28^ Further, clinicians in the current study enrolled patients with refractory pain not responding to medication. They ruled out improvements that could have been caused by other treatments and had no reason to view EM in a way that would bias their judgment as to clinical changes associated with the EM session.

## Conclusion

The authors found the results of this feasibility study encouraging regarding the acceptability, demand, and implementation of energy medicine in an inner-city community hospital setting. The practicality of carrying out a study with a single volunteer practitioner was good, albeit different from implementing an ongoing program that needs to be supervised by hospital staff. The next step regarding feasibility could be a study that explores methods for finding and screening local EM solo practitioners (whether volunteer or paid) and for integrating them into conventional clinical settings.

This study provides some guidance as to how EM can be applied clinically, especially in inpatient settings. It suggests that EM has a beneficial effect in some patients and provides some methodologic information that could be used in the design of stronger studies, such as funded feasibility studies of the integration of EM into conventional clinical settings.
